# Response of local dairy cows on lipid modulation in different temperature–humidity index (THI) zone

**DOI:** 10.5455/javar.2025.l929

**Published:** 2025-06-11

**Authors:** Didin Supriat Tasripin, Ujang Hidayat Tanuwiria, Andi Mushawwir, Iin Susilawati

**Affiliations:** Animal Husbandry Faculty, Universitas Padjadjaran, Jatinangor, Indonesia

**Keywords:** Altitude, dairy cattle, metabolism, lipid

## Abstract

**Objective:**

This study aimed to assess the impact of different rearing site zones with varying temperature–humidity index (THI) on the metabolic regulation of lactating local dairy cows.

**Materials and Methods:**

Forty local dairy cows were used in this study, consisting of 20 in rearing sites with THI 66–70 (Pangalengan) and 78–82 (Sumedang), at 950 and 550 m above sea level, respectively. Basal rations were given every morning and evening, consisting of forage and concentrate. Temperature and humidity were recorded daily to determine the average daily THI. Blood samples in both groups of experimental animals were collected according to standard procedures every month during the 4 months of the experiment. Blood analysis followed procedures based on protocols from KIT Randox (UK), using a spectrophotometer.

**Results:**

Lipid activity and regulation appeared higher (*p* < 0.05) in the group of lactating dairy cows kept at THI comfort zone 66–70 than at THI slight stress zone (78–82). Similarly, blood lipid levels were better (*p* < 0.05) in the cows in the comfort zone (66–70).

**Conclusion:**

The study’s results on the impact of the rearing zone of lactating local dairy cows appeared to affect the modulation of lipids in the body. Lipogenesis regulation and metabolism showed higher activity in the group of dairy cows reared in the comfort zone (THI = 66–70) compared to the group of dairy cows reared in the discomfort zone.

## Introduction

One of the dairy cattle breeds widely kept in Indonesia is the Friesian Holstein (FH) dairy cow. FH cattle milk production is high, reaching 5,982 kg per lactation with an average milk fat content of 3.7% [1], and has good adaptability to tropical and subtropical regions [2,3]. FH cows can produce optimal productivity if kept in environmental conditions with temperatures around 18°C and 55% humidity [4]. The ecological conditions of dairy cattle rearing, especially in West Java, are a problem related to temperature and moisture, even though the location is in the highlands. Research results [5,6] show that the average temperature of dairy cattle rearing locations in the highlands (>800 asl) is 25°C with 82% humidity.

FH dairy cows are homeothermic animals that require an optimum environmental temperature to live comfortably and produce [7,8]. The comfort zone for European dairy cows ranges from 13°C to 18°C [5,9]. Indonesia, especially in the Tunas Mekar KSU Tandangsari Livestock Group area, has an ambient temperature range of 25℃–31℃ and air humidity of 85% [4,10]. Climate differences in this maintenance can affect the physiological conditions and productivity of livestock, so it needs to be monitored for the survival of animals.

Keeping dairy cows above the comfort zone can cause heat stress that negatively affects physiological and hematological blood levels [11], changes in total leukocytes and leukocyte differentials, and dairy cows’ immune systems and inflammatory responses [12]. Leukocyte levels, including neutrophils, lymphocytes, monocytes, eosinophils, and basophils in the blood, are an indicator in evaluating heat stress experienced by dairy cows. In addition, heat stress can also lead to imbalances in physiological and metabolic functions. Excessive organ work mechanisms result in increased inflammation, damage, and cell death, which affects metabolite compounds in the blood such as total bilirubin, alkaline phosphatase, creatinine, creatine kinase [13,14], gamma-glutamyl transpeptidase, and lactate dehydrogenase that migrate to the blood circulation system, which means that the higher the levels of these compounds in the plasma, the more the cattle are experiencing heat stress [15].

The good adaptability of FH cows is indicated by good physiologic and production conditions, although still lower than in their country of origin. The low milk production produced can be influenced by the quality and quantity of feed given [16,17], so the nutrient needs of dairy cows are not met, which causes low milk biosynthesis [18]. Adding feed supplements to the ration aims to increase digestive efficiency so that physiological conditions [19,20] and livestock productivity increase [21,22].

Many studies have been reported, showing efforts to reduce the adverse effects of environmental factors on dairy cows caused by maintenance above the comfort zone, among others, through nutritional engineering such as providing feed supplements in the form of bypass protein [23], Ca-polyunsaturated fatty acid (PUFA) [10], and organic minerals [24]. These three types of feed supplements can reduce the adverse effects of livestock physiological conditions by stimulating metabolic rates [4,25] associated with lipid synthesis [26], increasing immunity [14], preventing inflammation, and supporting thermoregulation [6,13,27]. Some other researchers reported that feed protein tends to be degraded by rumen microbes to produce large amounts of ammonia [28,29]. Additional bypass protein is required through feed supplements. Bypass protein is a protein that rumen microbes cannot degrade. Therefore, most of it will be distributed to post-protein because post-rumen protease enzymes quickly digest this protein and have a high digestibility efficiency [30,31]. Previous research reports also show that feeding PUFA increases hematological levels of hematocrit, hemoglobin, and erythrocytes. PUFA (linoleic) compounds in feed are one of the precursors of prostaglandin hormones, one of whose function is to repair cells in the body, including erythrocyte cell membranes [6,16].

However, production efficiency and optimization through various feed engineering methods have not shown satisfactory results. One aspect that needs to be controlled is the drum’s microenvironment. The microenvironment of the drum diet plays a vital role in the balance of temperature and humidity. THI affects overall metabolism, so its approach to the appropriate diet must be studied carefully. Therefore, it is essential to observe their lipid metabolism, especially in lactating local dairy cows. Lipid metabolism plays a critical role in increasing milk production.

## Materials and Methods

### Ethical approval

This experiment was carried out and has been carefully reviewed by the Ethical Assessment Commission for Animal Experiments, Directorate of Research and Technology, DRT, with number 1027/Anim_Res.ER/11/23, to ensure that it meets the ethical requirements of animal experiments.

### Animal, housing, and location

The experimental animals used for this study were forty lactating FH dairy cows with 1st, 2nd, and 3rd lactation periods and 1st–5th lactation months and milk production of 8–12 l/day. The experimental cattle were given basal rations (grass and concentrate). The experimental animals were placed in individual pens measuring 1.5 × 2 m, each pen equipped with feed and water containers. Feeding was done in the morning and evening, while drinking water was given ad libitum.

This study was conducted at THI 66–70 and 78–82 rearing sites in Pangalengen, Bandung, and Tanjungsari, Sumedang. The altitudes of each area are 950 and 550 m above sea level. Daily temperature and humidity were recorded before and during the experiment.

### Data and blood sample collection

Blood samples were collected four times at weeks 5, 9, 12, and 16, respectively. Blood samples were taken from forty local dairy cows using a syringe needle in the jugular vein on the right side of the neck. As much as 5 ml of blood was collected in tubes containing ethylenediaminetetraacetic acid anticoagulant to obtain whole blood. The blood samples were then taken to the laboratory using a cooling box to prevent damage.

The blood samples were centrifuged for 15 min to separate the plasma. The blood plasma was analyzed using the spectrophotometer technique by mixing reagents and buffer solutions based on the Randox Kit instructions, UK, with wavelengths according to the respective parameters.

### Data analysis

Data were analyzed using an unpaired T-Student test analysis. Differences between treatments were determined using Mann–Whitney analysis/test. All analysis procedures were performed using SPSS IBM 25 software, with a significance level of 95%.

## Results and Discussion

### Lipid transport in plasma

Based on the current study’s results, Table 1 shows the concentrations of lipoproteins associated with lipid transport in the blood plasma of local dairy cows reared in terrestrial zones with different THI.

**Table 1.** The effect of THI on lipid transport in local dairy cows.

Parameters	Average THI during study (zone)	
	66–70 (Comfort zone)	78–82 (Minor stress zone)
Albumin lipid trans (μg/ml)	19.03 ± 0.23^a^	13.94 ± 0.25^b^
ApL A-I (gm/m)	3.18 ± 0.19^a^	2.04 ± 0.14^b^
ApL A-II (gm/l)	4.81 ± 0.89^a^	2.14 ± 0.78^b^
ApL B (gm/l)	5.93 ± 0,08^a^	1.12 ± 0.07^b^
ApL C-II (gm/l)	3.86 ± 0.11^a^	2.46 ± 0.09^b^
ApL C-III (gm/l)	4.63 ± 0.59^a^	1.63 ± 0.60^b^
ApL E (gm/l)	3.98 ± 0.08^a^	2.52 ± 0.07^b^
Cholesterol HDL (mg/dl)	97.55 ± 3.04^a^	78.78 ± 3.05^b^
Cholesterol LDL (mg/dl)	192.63 ± 4.06^a^	109.53 ± 3.95^b^
FATP1	14.62 ± 0.62^a^	9.24 ± 0.53^b^

Value in this table are means ± SD; a,b The average in the same line, marked with different superscripts, shows a significant difference (*p* < 0.05).

The current study results indicate that the rearing zones with different THIs overall increased the lipid transport activity in the blood plasma. This increase in transport activity was particularly evident in the group of local dairy cows reared in the comfort zone, with an average THI of 66–70 during the study. Table 1 shows that trans lipid albumin activity and low-density lipoprotein (LDL) cholesterol were highest in the group of cows in the comfort zone, at 19.03 μg/ml and 192.63 mg/dl, respectively.

Lipid transport activity in both groups of dairy cows reared in areas with different zones through venous and arterial vessels for inter-tissue purposes does not seem to be the same; this fact is characterized by higher levels of apolipoprotein (apl) A, Apl B, and Apl C (*p* < 0.05) in the group of dairy cows in the comfort zone (THI = 66–70). This result also proves that lipogenesis activity in the context of milk biosynthesis appears to be higher in dairy cows with a more comfortable environment.

It is known that ApL A and ApL B are instrumental in maintaining cholesterol concentration [26] and its biological function [4,12]. Apl A activity stimulates HDL production [3,25]. Increased HDL concentration increases lipid transport from tissues to liver cells [13,14]. Conversely, an increase in ApL B stimulates an increase in LDL, thereby increasing cholesterol transport from liver cells to tissues. During lactation, activity is needed to maintain the concentration of lipids [17,19] and cholesterol in the milk, as well as to maintain the concentration of lipid precursors in the alveoli cells [18]. Previous studies have also shown that feeding organic fatty acids and minerals can improve the physiologic condition of lactating cows [11], as well as maintain lipid homeostasis [9,12,32], and increase milk biosynthesis [18].

Table 2 shows the study’s results, based on which the effect of THI on the lipid profile of local dairy cow blood plasma is apparent. Based on this study, dairy cows’ blood lipid profile [cholesterol, non-esterified fatty acid (NEFA), and triglyceride (TAG)] in the comfort zone appeared to be higher than those of cows in the mild stress zone. Research [10,13] shows that comfortable environmental conditions for lactating dairy cows significantly increased the rate of lipogenesis, decreased the rate of adipocyte hypertrophy, increased TAG accumulation [14,15], increased lipoprotein lipase activity [9,13,16,26] in epididymal adipose tissue, and increased the activity of enzymes in the liver related to lipid biosynthesis [10,17,25].

**Table 2.** Effect of THI on plasma lipid.

Parameters	Average THI during study (zone)	
	66–70 (Comfort Zone)	78–82 (Minor Stress Zone)
Triglyceride (mg/dl)	173.12 ± 1.57^a^	152.83 ± 3.53^b^
NEFA (mg/l)	87.68 ± 2.63^a^	50.01 ± 2.63^b^
Cholesterol (mg/dl)	154.38 ± 3.36^a^	122.52 ± 3.76^b^
Glycerol (mg/dl)	32.73 ± 2.53^a^	19.68 ± 2.82^b^
C12:0 (% of total fatty acid)	0.98 ± 0.01^a^	0.84 ± 0.02^a^
C15:0 (% of total fatty acid)	0.53 ± 0.01^a^	0.47 ± 0.03^a^
C16:0 (% of total fatty acid)	18.34 ± 1.02^a^	20.53 ± 1.61^b^
C17:0 (% of total fatty acid)	1.82 ± 0.01^a^	1.55 ± 0.04^b^
C17:1 (% of total fatty acid)	1.43 ± 0.02^a^	0.35 ± 0.01^b^
C18:0 (% of total fatty acid)	12.42 ± 2.14^a^	9.42 ± 1.27^b^
C18:1 (% of total fatty acid)	38.93 ± 2.41^a^	32.25 ± 2.41^b^
C18:2 (% of total fatty acid)	6.82 ± 0.36^a^	4.36 ± 0.12^b^
C20:0 (% of total fatty acid)	4.63 ± 0.02^a^	2.52 ± 0.12^b^
C20:4 (% of total fatty acid)	5.53 ± 0.11^a^	4.17 ± 0.11^b^
C22:0 (% of total fatty acid)	4.53 ± 0.14^a^	2.63 ± 0.01^b^

Value in this table are means ± SD; a,b The average in the same line, marked with different superscripts, shows a significant difference (*p* < 0.050).

Comfortable conditions have been reported to activate adenosine monophosphate (AMP) significantly activated protein kinase (AMPK) phosphorylation [18,19,27]. A high-fat diet induces the expression of lipogenic transcription factors [Peroxisome proliferator-activated receptor gamma PPAR-γ and sterol receptor element-binding protein 1c] in the liver and adipose tissue [20,25,29].

The high cholesterol concentration in liver cells is one cause of the inhibition of 3-hydroxymethyl glutaryl coenzyme A (HMG-CoA) synthesis. This enzyme plays a role in cholesterol synthesis [11,16,21], so a decrease in its expression reduces endogenous cholesterol synthesis, and the amount of cholesterol circulating in the vascular system also decreases.

In addition, rearing conditions with ideal microenvironmental conditions appear to significantly increase the synthesis of PUFAs (Table 2). These conditions simultaneously inhibit the expression of lipogenic genes, including fatty acid synthase, HMG-CoA, fatty acid transport 1 (FATP1), and fatty acid binding protein 4 in the liver to synthesize saturated fatty acids [4,22,30]. Previous animal studies have also shown that the adipose tissue of experimental animals fed a diet high in unsaturated fats reduces saturated fat levels in the blood plasma [24,32].

The highest TAG level was 173.12 mg/dl in the comfort zone group, compared to the other groups. When related to research [25], the ideal THI factor significantly increases the rate of lipogenesis, decreases the rate of adipocyte hypertrophy, TAG accumulation, and lipoprotein lipase activity in epididymal adipose tissue [11,26], and reduces the activity of enzymes in the liver related to lipid biosynthesis [17,27].

Conversely, the discomfort zone causes an increase in lipolysis. The high lipolysis rate causes energy reserves, such as fat, to be broken down into NEFA and glycerol. NEFA is also a precursor for forming TAGs in adipose tissue, liver, and muscle through esterification [15,28,29]. However, in this study, high NEFA levels in the comfort zone, a sign that they are not formed into triacylglycerol/ TAG, are due to NEFA contributing to the formation of milk lipids.

### Regulation of peroxisome proliferator-activated receptor gamma on total lipid

**Figure 1.**
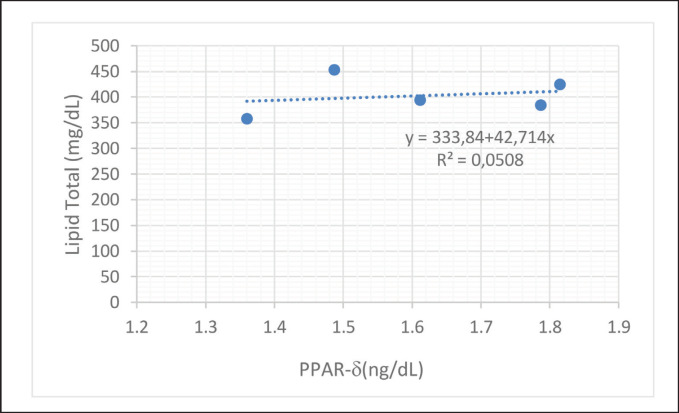
Prediction model and effect of PPAR-γ on total lipids of dairy cows at rearing sites with average THI 78–82.

**Figure 2.**
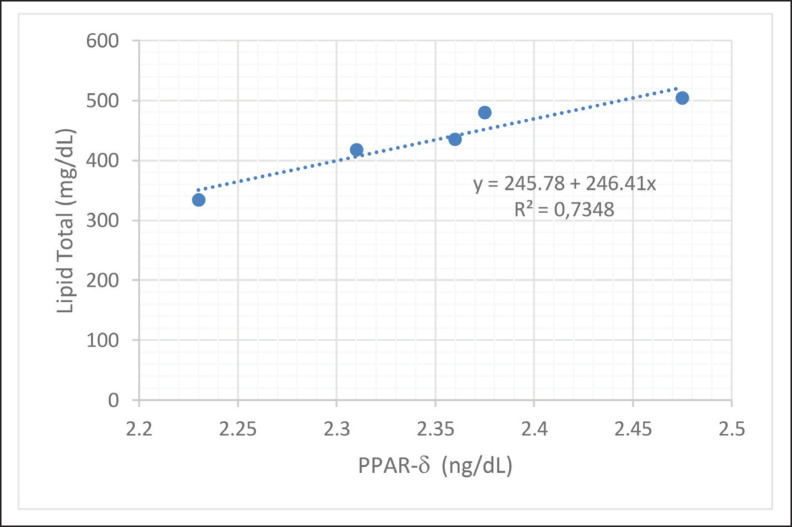
Prediction model and effect of PPAR-γ on total lipids of dairy cows at rearing sites with average THI 66–70.

The influence of PPAR protein on total plasma lipids in dairy cows reared with THI 78–82 (Fig. 1) was 0.5%, and a more significant impact, 73.48%, was shown in dairy cows reared in the comfort zone (Fig. 2).

PPAR-g are proteins that regulate lipid homeostasis in the body. Several previous studies have reported that lipid regulation and modulation in cells and tissues is determined by PPAR-gene expression. Increased gene expression correlates positively with cell and plasma lipid concentration balance [4,29]. Several other studies have also shown the critical role of PPAR-g in maintaining blood cholesterol [20], lipids in cells [15,30], and lipid transport from and to tissues [3,19,31].

Feed supplementation at low altitudes was shown to stimulate PPAR-gene expression; therefore, increasing the concentration of this gene significantly increased lipid concentration. Previous researchers [5,6,32] have also reported similar results, showing that total lipids can be controlled by administering protein and essential fatty acids through PPAR and sterol binding protein receptor-1α [11,30,32].

## Conclusion

The study’s results on the impact of the rearing zone on lactating local dairy cows appear to affect lipid modulation in the body. Regulation and metabolism of lipogenesis showed higher activity in dairy cows kept in the comfort zone (THI = 66–70) than in the less comfortable zone (THI = 78–82). PPAR-g regulator also showed a higher influence on dairy cows in comfort conditions.

## List of abbreviations

AMP, adenosine monophosphate; Apl, apolipoprotein; FATP1, fatty acid transport 1; FH, Frisien Holland; HMCCOA, 3-hydroxyl-3-methyl-glutaryl Co-A reductase; LDL, low density lipoprotein; NEFA, non-esterified fatty acid; PPAR-γ, peroxisome proliferator-activated receptor gamma; PUFA, polyunsaturated fatty acid; TAG, triglycerides; THI, temperature–humidity index.
